# Effects of jasmonic acid in foliar spray and an humic acid amendment to saline soils on forage sorghum plants’ growth and antioxidant defense system

**DOI:** 10.7717/peerj.13793

**Published:** 2022-10-14

**Authors:** Adam Yousif Adam Ali, Guisheng Zhou, Aboagla Mohammed Elsiddig, Guanglong Zhu, Tianyao Meng, Xiurong Jiao, Irshad Ahmed, Ebtehal Gabralla Ibrahim Salih, Muhi Eldeen Hussien Ibrahim

**Affiliations:** 1 Department of Agronomy, Faculty of Agricultural and Environmental Science, University of Gadarif, Al Gadarif, Sudan; 2 Joint International Research Laboratory of Agriculture and Agri-Product Safety of the Ministry of Education of China, Yangzhou University, Yangzhou City, China; 3 Faculty of Forestry, University of Khartoum, Khartoum, Sudan; 4 Department of Agronomy, College of Agricultural Studies, Sudan University of Science and Technology, Khartoum, Sudan

**Keywords:** Abiotic stress, Salinity, Jasmonic acid, Humic acid, Forage sorghum, Antioxidant defense system, Plant growth and development, Reactive oxygen species, Physiological parameters, Plant hormone

## Abstract

Salinity is one of the primary abiotic stresses that cause negative physiological and biochemical changes due to the oxidative stress caused by the generation of reactive oxygen species (ROS). The effect of jasmonic acid (JA) as foliar spray and humic acid (HA) as soil amendment on the growth and biochemical attributes of forage sorghum plants exposed to salinity stress was investigated. Soil treated with NaCl at levels of 0, 2, and 4 g NaCl kg^−1^ dry soil (designated as S0, S1, and S2) and soil amendment with humic acid at 0, 3, and 6 g HA kg^−1^ dry soil (designated as HA0, HA1, and HA2). The plants were sprayed with three JA levels, including 0, 5, and 10 mM JA. Salinity stress increased carotenoid and soluble protein content, superoxide dismutase (SOD) activity, and malondialdehyde (MDA) content. In contrast, salinity stress reduced plant height, leaf area, relative growth rate, proline content, and the activity of peroxidase (POD), catalase (CAT), and ascorbate peroxidase (APX). At the S2 salinity level, HA2 rate increased plant high by 9.7%, relative growth rate by 70.8% and CAT by 45.5, while HA1 increased leaf area by 12.5%, chlorophyll content by 22.3%, carotenoid content by 38.1%, SOD activity by 20.9%, MDA content by 18.0%, POD activity by 24.6% and APX value by 21.7%. At the S2 salinity level, the highest plant height, chlorophyll content, soluble protein content and APX value were recorded at 5 mM JA, while the highest leaf area, the content of carotenoid, proline, and MDA, and the activity of POD and CAT were achieved at 10 mM JA. Generally, 10 mM JA and 3 g HA kg^−1^ dry soil produced the best positive effects on forage sorghum plants physiological responses. Our study suggested that jasmonic acid and humic acid at appropriate rates can successfully mitigate the adverse effects of salinity stress on forage sorghum.

## Introduction

Salinity stress is one of the abiotic stresses limiting crop growth and productivity. Under salinity stress, crop plants usually experience many profound changes in morphological and physiological processes (Hussien Ibrahim et al., 2019). Sodium chloride is essential for structural and functional parts of the vital machinery of plant cells. However, this requirement is very low for normal growth and development of crop plants (Ali et al., 2021). Unfortunately, plants under saline stress often have an oversupply and accumulation of NaCl through their roots, triggering specific physiological responses (Badawy et al., 2021). Salinity stress can affect at all plant growth stages. The root zone is known to be more sensitive to salinity, causing significant inhibittion of root elongation, and ultimately reducing crop yield due to osmotic stress, ion toxicity, and reduced absorption of essential nutrients such as Ca^+2^ and K^+^ (Farhangi & Ghassemi, 2018).

The over accumulation of reactive oxygen species (ROS) are harmful to plant growth and development as it affects the structure and functions of biomolecules (Shafiq et al., 2014; Ahmad et al., 2016). Antioxidant enzymes such as catalase (CAT), peroxidase (POD), and superoxide dismutase (SOD) usually remove H_2_O_2_ to hydrogen peroxide and dioxygen (Dustgeer et al., 2021). The activities of SOD, CAT, and POD can increase during biotic and abiotic stresses to protect cells from potentially hazardous effects of ROS (Menezes et al., 2004). CAT is localized in leaf tissue in peroxisomes to scavenge the H_2_O_2_ produced by glycolate oxidase (Shafiq et al., 2014).

Jasmonates (JAs), including methyl jasmonate (MeJA) and jasmonic acid (JA) can stimulate MDA accumulation and inhibit Fe-induced release of chelators that counteract salt stress. It is suggested that MeJA triggers some other protective mechanisms (Kumari & Sudhakar, 2003). Jasmonic acid (JA) is a lipid-derived plant hormone that mediates diverse biological phenomena and is a critical regulator of plant responses to salinity (Ali et al., 2019, 2020). It is a member of plant growth regulators, which are important cellular regulators involved in many developmental processes such as germination, root growth, and fertility (Sofy et al., 2020). Rakwal, Agrawal & Yonekura (1999) observed that high JA level in wounded leaves caused significant changes in the protein pattern of rice plants. Exogenous JA application can increase and regulate antioxidant activities in different plants (Shafiq et al., 2014). dos Santos Soares et al. (2010) noticed a gradual accumulation of H_2_O_2_ in *Ricinus communis* (L.) and a sharp formation of ROS at the initial moment of MeJA and ascribed it to the decrease in the enzymatic antioxidants activities. In another study with pomegranate (*Punica granatum* L.), Vatanparast, Mirdehghan & Karimi (2013) observed an increase in antioxidant activity in the foliage treated with JAs. Wolucka, Goossens & Inzé (2005) found that in the treatment of plants with JAs application, APX activity was upregulated in tobacco (*Nicotiana tabacum* L.) plants.

Humic acid (HA) is an organically charged bio-stimulant that significantly impacts plant growth and development (Ali et al., 2020). HA can alleviate salt stress through improving plant growth, maintaining water potential and increasing crop productivity (Ebrahimi & Miri, 2016). Xueyuan et al. (2001) found that low level of HA significantly increased wheat plant growth (*Triticum aestivum* L.). Kaya et al. (2018) showed that HA application led to increased stem and root dry weight of corn plants (*Zea mays* L.). Notably, humic acid can mitigate different stresses by increasing dry biomass weight and promoting plant growth (Kaya et al., 2018).

Sorghum (*Sorghum bicolor* (L.) Moench) is one of the most important high-productive cereal crops (Sun, Shi & Ding, 2017). It has become one of the most efficient sources of food and feed. Additionally, it is used for bioenergy production (Seleiman et al., 2021). It can adapt to diverse environmental conditions, especially in arid and semi-arid areas (Mishra, Kumar & Rao, 2017). Sorghum is a C4 plant that is graded having moderate tolerance to soil salinity. However, its growth and yield is significantly reduced when cultivated under salinity stress (Maswada, Djanaguiraman & Prasad, 2018).

Due to the progressive salinization of world arable lands, the practice of using exogenous hormone protectants to mitigate salt-induced damages has been more important than ever. However, to our knowledge, there are not adequate reports on the effects of exogenous application of JA, soil amendment with HA, and their combination in antioxidant system regulation, photosynthetic pigments, soluble protein, and proline content of forage sorghum plants subject to salinity. We hypothesized that phytohormones such as JA, soil amendment with HA, and their combination, could play a vital role in plant tolerance toward salinity stress by boosting plant growth, antioxidant system regulation, and photosynthetic of pigments of sorghum plants.

## Materials and Methods

### Location and climate of the experimental site

A 2-year pot experiment was conducted in an unheated greenhouse in the two consecutive years of 2018 and 2019 at Joint International Research Laboratory of Agriculture and Agri-Product of Ministry of Education of China, Yangzhou University, Yangzhou, Jiangsu Province, China (32°39′N, 119°41′E). During the experiment, the average temperature was 31 °C, relative humidity was 76%, and cloud cover was 40%. Peat moss and vermiculite mixture (1:2 v/v) was used as the germinating and growing media of sorghum plants.

### Soil characteristics

The soil was collected from the surface of sandy loam soil (0–20 cm) of the Experimental Farm of Yangzhou University. The soil was presented as a sandy loam texture. The chemical and physical properties of the potting soil and chemical properties of HA are presented in Table 1. The soil was air-dried and passed through a 5 mm mesh screen. The soil was then spread over a piece of polyethylene sheet at a thickness of about 70 mm. The soil suspension was prepared in deionized water at a ratio of 1:2 (w/w) soil:water. The suspension was shaken and allowed to stand overnight. After that, the electrical conductivity of the supernatant solution was determined at 0.26 dSm^−1^ using a conductivity meter (TZS-EC-I; Zhejiang Top Instrument Co., Ltd., Hangzhou, China).

**Table 1:** Analysis of variance of two season. Chemical and physical properties of soil and humic acid used in this study.

Characters		Soil	Humic acid
pH		7.1	7.08
EC (dS.m^−1^)		1.2	--------
CEC (c.molc kg^−1^)		12.0	--------
Organic matter (%)		12.2	55
Available macronutrients	N (mg kg^−1^)	94.8	4.87
	P (mg kg^−1^)	14.1	0.01
	K (mg kg^−1^)	77.3	11.21
Soluble ions	Ca (meq L^−1^)	18.84	0.50
	Mg (meq L^−1^)	17.20	0.22

### Plant material

The seeds of forage sorghum variety Abu Sabeein were obtained from Agricultural Research Corporation, Khartoum, Sudan. The seeds were less than 8 months old and had been stored in paper bags under laboratory conditions (RH 40–60% at 15–20 °C). Seeds were surface-sterilized with 3% sodium hypochlorite solution for 1 min and then thoroughly rinsed three times with deionized water and air-dried near to their original weight for seeding. Seeds were germinated on 10^th^ May in 2018, and on 15^th^ May in 2019 in a seedbed for 15 days in the greenhouse. The strongest and uniform seedlings were selected and transplanted into pots. Each pot (30 cm in diameter × 32 cm in depth) was filled with 15 kg dry soil. On the 15^th^ day after seedlings were transferred and applied with the NPK fertilizer (50 kg N/ha + 50 kg P2O5/ha + 50 kg K2O/ha). Another half dose was used on the 45^th^ days of transplanting, and according to local recommendations, a spray of pesticides and weed control were conducted. Furthermore, the plants were watered regularly to maintain the water level every 3 days with a tap water.

All required approvals were obtained for the study, which complied with all relevant regulations.

### Experimental design and treatments

This study was a three-factorial experiment arranged in a split–split-plot randomized complete block design with three replications. The main plots included three salinity levels at 0 (S0), 2 (S1), and 4 g NaCl kg^−1^ dry soil (S2) (with an equivalent EC of 0.26, 2.3, and 4.7 dS m^−1^,respectively). The subplots included three rates of humic acid, including 0, 3 and 6 g HA kg^−1^ dry soil, designed as HA0, HA1 and HA2, respectively. The sub–sub-plot included three levels of jasmonic acid, including 0, 5, and 10 mM JA.

Before seedling transplant, humic acid and NaCl at different levels were thoroughly mixed with the soil to make nine different treatments of salinity and HA. Before salinity and humic acid treatment, a 100 g soil sample was collected and oven-dried at 75 °C to constant weight, and the moisture content was calculated. On the 15^th^ day after seedling transplant, the plants at each NaCl level and humic acid rates were treated with exogenous jasmonic acid solutions as a foliar application. The spraying was repeated every 15^th^ day. The plants were sprayed three times. During jasmonic acid application, care was taken to avoid any drift of different levels using a plastic shelter to separate each treatment. There were 27 treatments in the study with three replicates for each treatment. There were 81 pots, and all pots were placed in a greenhouse. Tap water (EC 0.26 dSm^−1^) was used. Pots were weighed every 2 or 3 days to maintain soil water content at 80% field capacity. The pots had no holes at the bottom to prevent drainage and leaching.

### Observations and measurements

#### Plant height, leaf area, and relative growth rate

Plant height (cm) was recorded on the 50^th^ (Time one) and 70^th^ (Time two) day after sowing. Three plants from every plot were randomly selected and tagged. Plant height was measured from a point immediately above the soil surface to the top of the plant. Four uppermost leaves including flag leaf from three plants of each pot were selected for measuring leaf area with a leaf area meter (LI-3100C; LI-COR Biosciences, Lincoln, NE, USA). The relative growth rate was calculated according to Hoffmann & Poorter (2002):

${\rm Relative\; Growth\; Rate} = \displaystyle{{{\rm Plant\; height\; at\; time\; two} - {\rm Plant\; height\; at\; time\; one}} \over {{\rm Time\; two} - {\rm Time\; one}}}$

#### Preparation of enzyme extracts

On the 50^th^ day of seedling transplant, a healthy leaf from the middle of the plant excluding the four uppermost leaves, was harvested and immersed in liquid nitrogen for 20 min and stored in a low-temperature freezer to determine the activity of enzymes and the content of proline and soluble protein. Leaf protein was extracted using a phosphate buffer solution containing sodium phosphate dibasic dehydrate and sodium phosphate monobasic dehydrate. Stored leaf tissue (0.2 g) was crushed in 2 ml of the phosphate buffer solution, and the slurry was centrifuged at 10,000*g* for 20 min at 4 °C. The supernatant was used for the determination of the activities of enzymes including SOD, CAT, POD, and APX.

#### Biochemical assays

The activity of SOD and CAT was determined following the method of Janmohammadi, Abbasi & Sabaghnia (2012). The POD activity was assayed according to the method of Xu & Ye (1989). The MDA content was determined following the method of Zhang et al. (2007). The APX content was measured according to Nakano & Asada (1981).

Soluble protein was estimated for each extract (Bradford, 1976). A dye stock solution was added to the earlier centrifuged samples and the samples were incubated at room temperature for 25–30 min. The absorbance of the reaction mixture was recorded at 595 nm.

Proline content was estimated using the protocol of Bates, Waldren & Teare (1973). Fresh sample (0.5 g) was extracted with sulfosalicylic acid, and the extract was filtered to separate the residue. All the filtrates were mixed with acidic ninhydrin, orthophosphoric acid, and glacial acetic acid and incubated at 100 °C for 30 min. The mixtures was cooled, incorporated in toluene, and vortexed. The absorbance of the reaction mixture was recorded at 520 nm with a spectrophotometer.

#### Photosynthetic pigments

The determination of photosynthetic pigments such as total chlorophyll content and carotenoid content were conducted according to the method reported by Lichtenthaler & Wellburn (1983). Each fresh leaf sample was soaked in acetone solution (80%) in the dark for 7 days. The absorbance readings were recorded at 453, 645, and 663 nm, respectively, using a spectrophotometer.

#### Statistical analysis

This study was performed in two different seasons, and there were no significant differences in all the parameters between the two seasons (Table 2). Therefore, the average of each variable of the two seasons was applied for statistical analysis. The data of each variable were subjected to analysis of variance (ANOVA) for factorial experiment arranged in a split–split-plot randomized complete block design with the statistical package of MSTAT-C (Freed et al., 1991). When *F* values were significant, means were separated by the least significant difference (LSD) test (*P* ≤ 0.05) as described by Snedecor & Cochran (1980). The standard error of the average were calculated for each trait.

**Table 2:** Analysis of variance. Analysis of variance for effects of jasmonic acid, humic acid, salinity and their interaction on growth parameters, chlorophyll content, soluble protein, proline content, and antioxidant enzymes of forage sorghum in two growing seasons (2017–2018 and 2018–2019).

Dependent variables	Independent variables (*F* value)							
	Season	Jasmonic acid (JA)	Salinity (S)	JA × S	Humic Acid (HA)	HA × S	HA × JA	JA × S × HA
Plant height	2018	357.6**	7.9**	21.6***	6.9**	9.6***	3.0*	3.8**
	2019	6.6*	68.1***	249.3***	22.8***	3.1*	4.7**	7.4***
Leaf area	2018	452.7***	13.3***	1.3^ns^	5.8**	1.4^ns^	6.8**	2.9*
	2019	1.93^ns^	31.1***	12.5***	136.0**	7.4**	24.2**	17.7**
Relative growth rate	2018	284.2**	17.0**	27.3**	3.7*	10.5**	1.2^ns^	3.3**
	2019	0.41^ns^	33.5**	74.7**	3.4*	1.7^ns^	2.1*	3.2**
Chlorophyll content	2018	23.9**	3.26*	2.1^ns^	4.9*	12.7**	7.4**	13.5**
	2019	8.22*	35.4***	4.22*	2.30^ns^	3.62*	0.52^ns^	1.14^ns^
Carotenoid content	2018	16.58*	25.88**	21.44**	81.80**	17.62**	52.72**	5.59^ns^
	2019	6.50*	10.04*	0.69^ns^	4.59*	7.94*	3.15*	3.43^ns^
Soluble protein	2018	3.14*	11.32**	2.89*	3.30*	2.28*	0.68*	2.99^ns^
	2019	52.03**	27.49**	3.30*	23.28*	1.54^ns^	1.67^ns^	1.59^ns^
Proline content	2018	121.51**	523.95**	47.22**	73.47*	38.27**	16.60*	27.41*
	2019	16.08*	1.67^ns^	1.52^ns^	4.60*	2.76*	1.41^ns^	3.07*
Superoxide dismutase (SOD)	2018	14.53*	0.75^ns^	1.47^ns^	8.83***	1.04^ns^	3.47*	6.64***
	2019	4.30^ns^	6.73*	4.61*	2.67*	3.08*	5.28**	5.36***
Peroxidase (POD)	2018	3.25^ns^	16.34***	2.28^ns^	6.30**	2.12*	3.73*	2.53*
	2019	1.39^ns^	19.70***	5.32*	2.22^ns^	4.04**	4.74**	3.11**
Catalase (CAT)	2018	5.81*	9.54**	12.34***	90.94***	3.92**	19.53**	9.06**
	2019	2.10^ns^	0.67^ns^	7.74**	32.12***	8.25**	4.82**	7.54**
Malondialdehyde content (MDA)	2018	42.07**	6.29*	8.75**	22.90**	10.25**	19.09**	16.29**
	2019	41.14**	7.47**	11.47**	3.49*	2.86*	2.67*	4.66**
Ascorbate peroxidase **(**APX)	2018	17.33*	12.86*	1.96^ns^	19.31**	2.62**	4.79**	3.55**
	2019	11.40*	56.50**	0.64^ns^	8.21**	4.97**	5.19**	6.90^ns^

**Notes:**

* Significant differences at *P* ≤ 0.05 probability level.

** Significant differences at *P* ≤ 0.01 probability level.

*** Significant differences at *P* ≤ 0.001 probability level.

ns, no significant difference.

## Results

The results revealed that jasmonic acid, humic acid, salinity, and their interactions significantly affected most parameters measured on most occasions (Tables 2 and 3).

**Table 3:** Interaction between salinity and humic acid on relative growth rate and APX. Analysis of variance table of the average of two seasons for the effects of jasmonic acid, humic acid, salinity and their interaction on growth parameters chlorophyll content, carotenoid contents, soluble protein, proline content, and antioxidant enzymes activities of forage sorghum.

	F value											
	Plant height	Leave area	Relative growth rate	Chlorophyll content	Carotenoid content	Soluble protein	Proline content	SOD	POD	CAT	MDA	APX
Salinity (S)	74.24***	66.87***	16.1***	20.10**	6.9*	7.97*	5.28*	1.56^ns^	22.44**	0.29^ns^	11.6*	25.9**
Humic acid (HA)	22.6***	120.7**	0.1^ns^	10.31**	12.3**	5.37*	12.56**	0.88^ns^	2.4^ns^	4.11*	48.0***	6.34*
S × HA	7.91**	10.95**	7.5***	5.99*	20.6***	2.36^ns^	2.18^ns^	4.06*	11.62**	6.44**	19.8**	2.6*
Jasmonic acid (JA)	342.2**	17.09*	12.1*	25.09**	80.5***	3.30*	14.83**	8.11**	2.3^ns^	40.96***	9.46*	23.8**
S × JA	246.0***	10.98***	4.6*	8.60*	41.3***	0.68^ns^	6.41**	1.74^ns^	3.32*	6.23***	13.1**	4.0**
HA × JA	5.37**	11.24^ns^	2.5*	10.11*	14.2***	2.28*	0.88^ns^	2.69*	2.21*	8.33***	2.44^ns^	6.5^ns^
S × HA × JA	0.85^ns^	1.90^ns^	0.9^ns^	1.14^ns^	3.2^ns^	0.99^ns^	0.37^ns^	1.67^ns^	0.95^ns^	0.63^ns^	8.21^ns^	0.84^ns^

**Notes:**

* Significant differences at *P* ≤ 0.05 probability level.

** Significant differences at *P* ≤ 0.01 probability level.

*** Significant differences at *P* ≤ 0.001 probability level.

ns, no significant difference; SOD, superoxide dismutase; POD, peroxidase; CAT, catalase; MDA, malondialdehyde content; and APX, ascorbate peroxidase.

### The growth parameters as affected by the combination between salinity and humic acid

**Figure 1:**
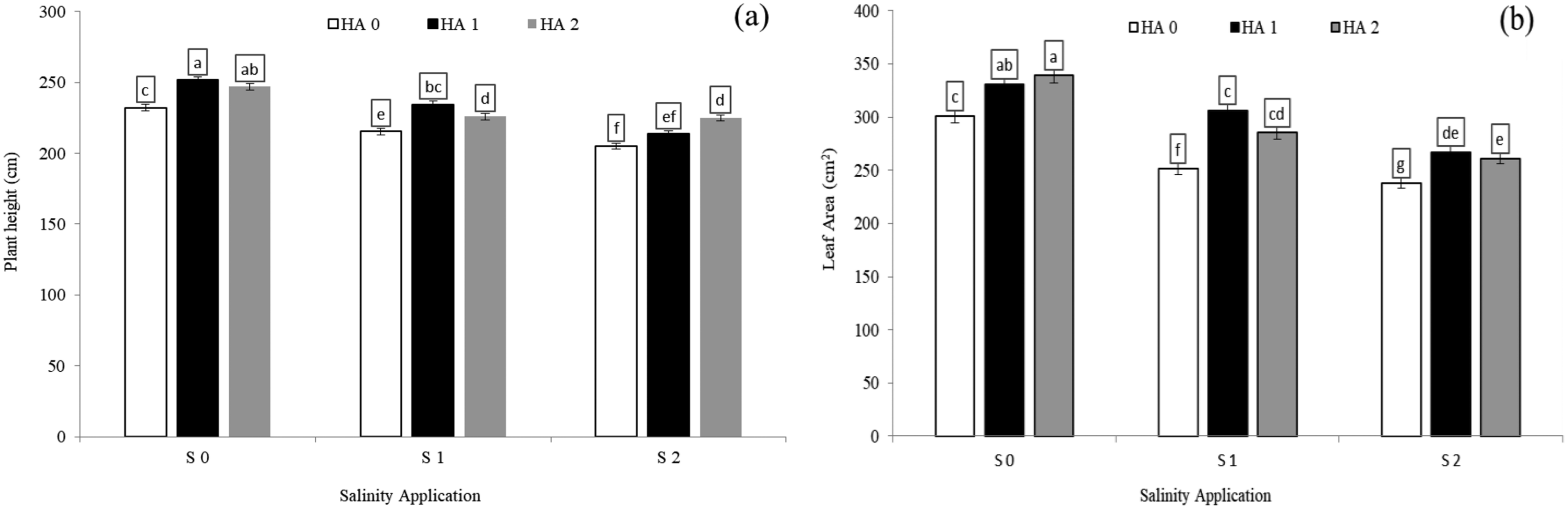
The interaction between salinity and humic acid on plant height and leave area. Plant height (A) and leaf area (B) of forage sorghum (*Sorghum bicolor* (L.) Moench) variety Abu Sabeein as effluence by the interaction between different salinity levels S0 = 0, S1 = 2 and S2 = 4 g NaCl kg^−1^ dry soil and different humic acid rates (HA0 = 0, HA1 = 3 and HA2 = 6 g HA kg^−1^ dry soil). Data were averaged over two growing seasons. The values of each trait labeled by different letters indicate significant differences separated by the LSD test (*P* < 0.05).

The growth parameters such as plant height, leaf areas and relative growth rate were significantly decreased with increased salinity rate. In the interaction between salinity and humic acid, at the control of humic acid (HA0), the high salinity rate of S2 reduced the plant height by 9.1% (Fig. 1A), leaf area by 20.0% (Fig. 1B), and relative growth rate by 44.7% (Table 4), in comparison with the control of salinity (S0) at the control of humic acid (HA0). The growth parameters were improved by humic acid. At the high salinity level of S2, the high rate of humic acid (HA2) were increased the plant height by 9.7% (Fig. 1A), and relative growth rate by 70.8% (Table 4), in comparison with control of humic acid of HA0 (Fig. 1A) at the same salinity level. At the same salinity level of S2, the rate of HA1 increased the leaf area by 12.45% (Fig. 1B), in comparison with HA0 (Fig. 1B) at the S2. At the medium salinity rate of S1, the treatment of HA1 achieved the highest level of plant height (Fig. 1A), leaf area (Fig. 1B), and relative growth rate (Table 4).

**Table 4:** Interaction between humic and salinity on plant height, relative growth rate, total chlorophyll content and carotenoid content. The average of two season of relative growth rate and ascorbate peroxidase (APX) activity of forage sorghum (*Sorghum bicolor* (L.) Moench) variety Abu Sabeein as effected by the interaction between different salinity levels (S0 (0 g NaCl kg^−1^ dry soil), S1 (2 g NaCl kg^−1^ dry soil) and S2 (4 g NaCl kg^−1^ dry soil)) and different humic acid rates (0 (HA0), 3 (HA1), and 6 g HA kg^−1^ dry soil (HA2)).

Salinity rates	Humic acid rates	Relative growth rate	APX (U g^−1^ min^−1^)
S0	HA0	3.65 ± 0.48cd	51.68 ± 18.53d
	HA1	4.45 ± 1.20ab	91.66 ± 38.52a
	HA2	4.65 ± 1.28a	77.81 ± 24.00b
S1	HA0	3.02 ± 0.81e	46.27 ± 8.87g
	HA1	4.27 ± 1.12b	67.96 ± 31.60e
	HA2	3.85 ± 0.81c	72.27 ± 19.70c
S3	HA0	2.02 ± 0.69f	39.72 ± 14.70h
	HA1	3.00 ± 1.19e	54.79 ± 12.13f
	HA2	3.45 ± 1.49d	33.35 ± 14.55i

**Note:**

All analyzed data are expressed as mean ± SD values of three biological replicates per treatment. Within the same parameter, means followed by different letters are statistically different at the 0.05 probability level. Means separated by the LSD test.

### The growth parameters as affected by the combination between salinity and jasmonic acid

**Figure 2:**
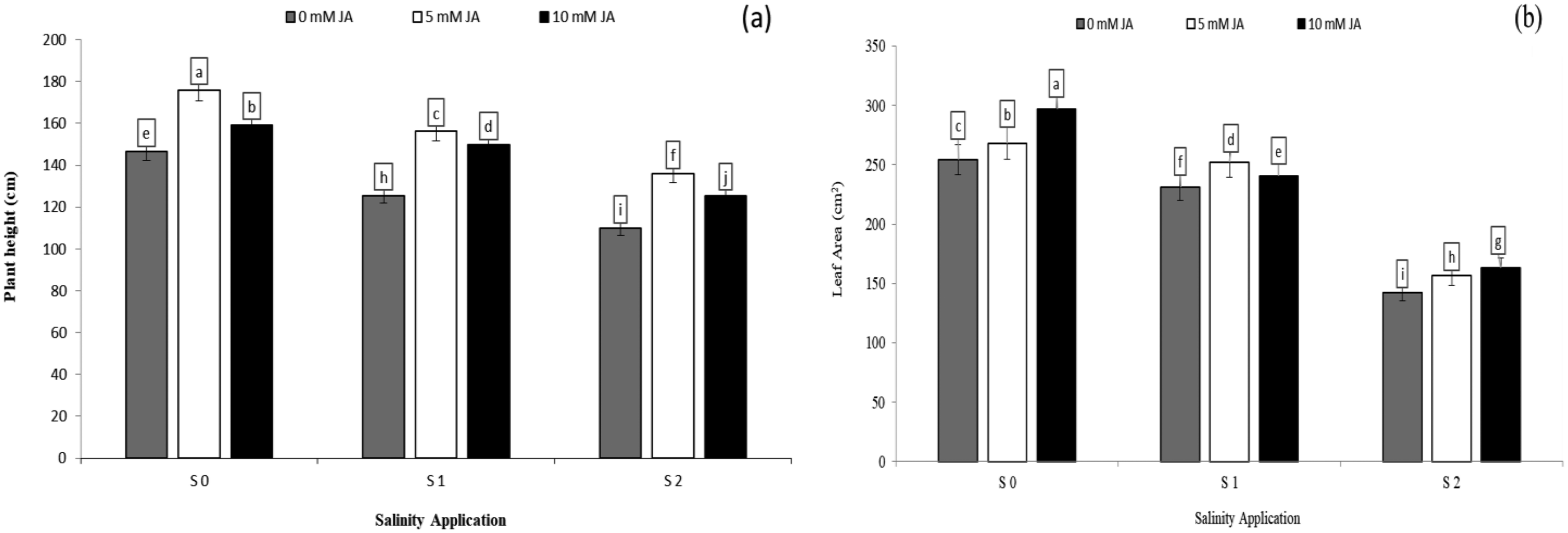
The interaction between salinity and jasmonic acid on plant height and leave area. Plant height (A) and leaf area (B) of forage sorghum (*Sorghum bicolor* (L.) Moench) variety Abu Sabeein as effluence by the interaction between different salinity levels S0 = 0, S1 = 2 and S2 = 4 g NaCl kg^−1^ dry soil and different jasmonic acid levels. Data were averaged over two growing seasons. The values of each trait labeled by different letters indicate significant differences separated by the LSD test (*P* < 0.05).

Regarding the combination between salinity and jasmonic acid, jasmonic acid improved the plant height, leaf area and relative growth rate. Both jasmonic acid levels increased all the growth parameters. According to the results of the interaction between salinity and jasmonic acid, at the high saline rates of S2, both jasmonic acid levels of 5 and 10 mM JA significantly increased the plant height by 23.63% and 14.20% respectively (Fig. 2A), and leaf area by 10.07% and 14.71% respectively (Fig. 2B), as compared with control of salinity (S0). Under the medium salinity rate of S1, the level of 5 mM JA increased the plant height from 125.6 to 156.2 cm (Fig. 2A), and leaf area from 231.3 to 251.9 cm^2^ (Fig. 2B).

### Photosynthetic pigments as affected by the combination between salinity and humic acid

**Figure 3:**
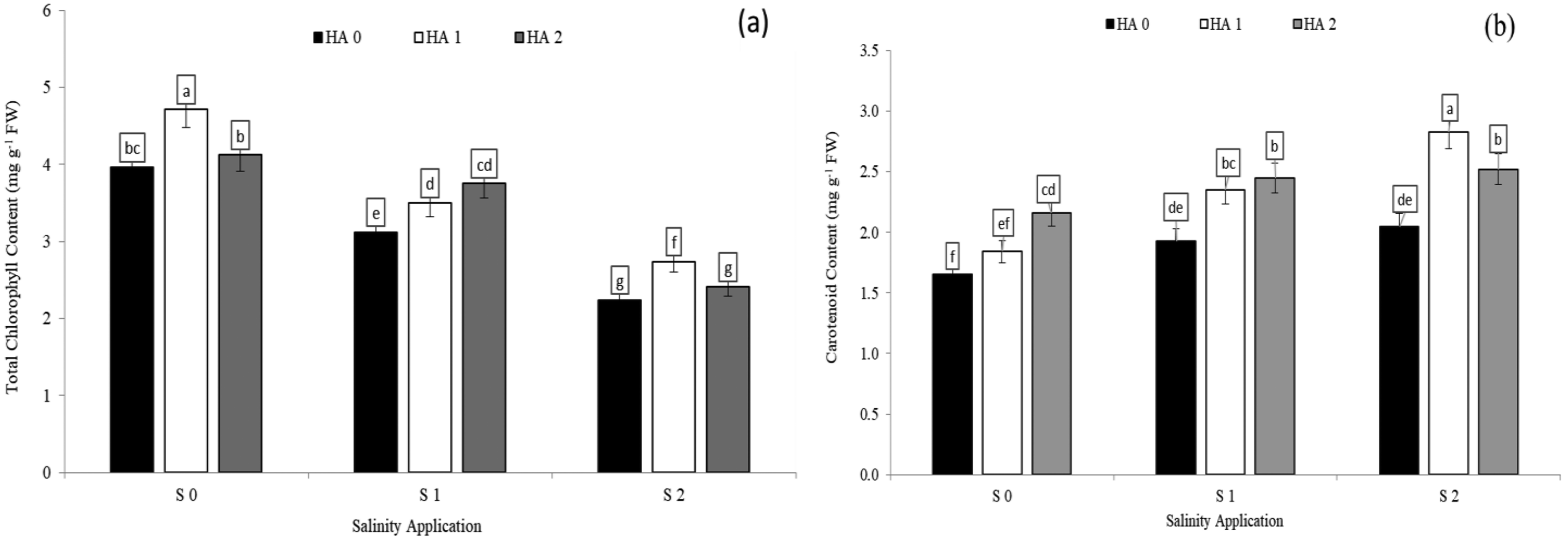
The interaction between salinity and humic acid on total chlorophyll content and carotenoid content. Total chlorophyll content (A) and carotenoid content (B) of forage sorghum (*Sorghum bicolor* (L.) Moench) variety Abu Sabeein as effluence by the interaction between different salinity levels S0 = 0, S1 = 2 and S2 = 4 g NaCl kg^−1^ dry soil and different humic acid rates (HA0 = 0, HA1 = 3 and HA2 = 6 g HA kg^−1^ dry soil). Data were averaged over two growing seasons. The values of each trait labeled by different letters indicate significant differences separated by the LSD test (*P* < 0.05).

Chlorophyll content was significantly decreased with increased soil salinity. However, increased salinity rate significantly increased carotenoid content. In the interaction between salinity and humic acid, at the control of humic acid (HA0), the high soil salinity rate of S2 decreased the chlorophyll content by 43.4% (Fig. 2A) in comparison with S0 at the same rate of humic acid (HA0). Moreover, at the same rate of humic acid (HA0), the high salinity rate of S2 recorded the highest carotenoid content (2.05 mg g^−1^ FW), while S0 (control of salinity) showed the lowest value (1.65 mg g^−1^ FW) (Fig. 2B). The chlorophyll content and carotenoid content were improved by humic acid application. At the high salinity rate of S2, the treatment of HA1 increased the chlorophyll content by 22.3% (Fig. 3A), and carotenoid content by 38.1% (Fig. 3B), in compared with control. At the rate of S1, the highest value of chlorophyll and carotenoid content were recorded at the treatment HA2 (Figs. 2A and 2B respectively).

### Photosynthetic pigments, soluble protein and proline content as affected by the combination between salinity and jasmonic acid

**Figure 4:**
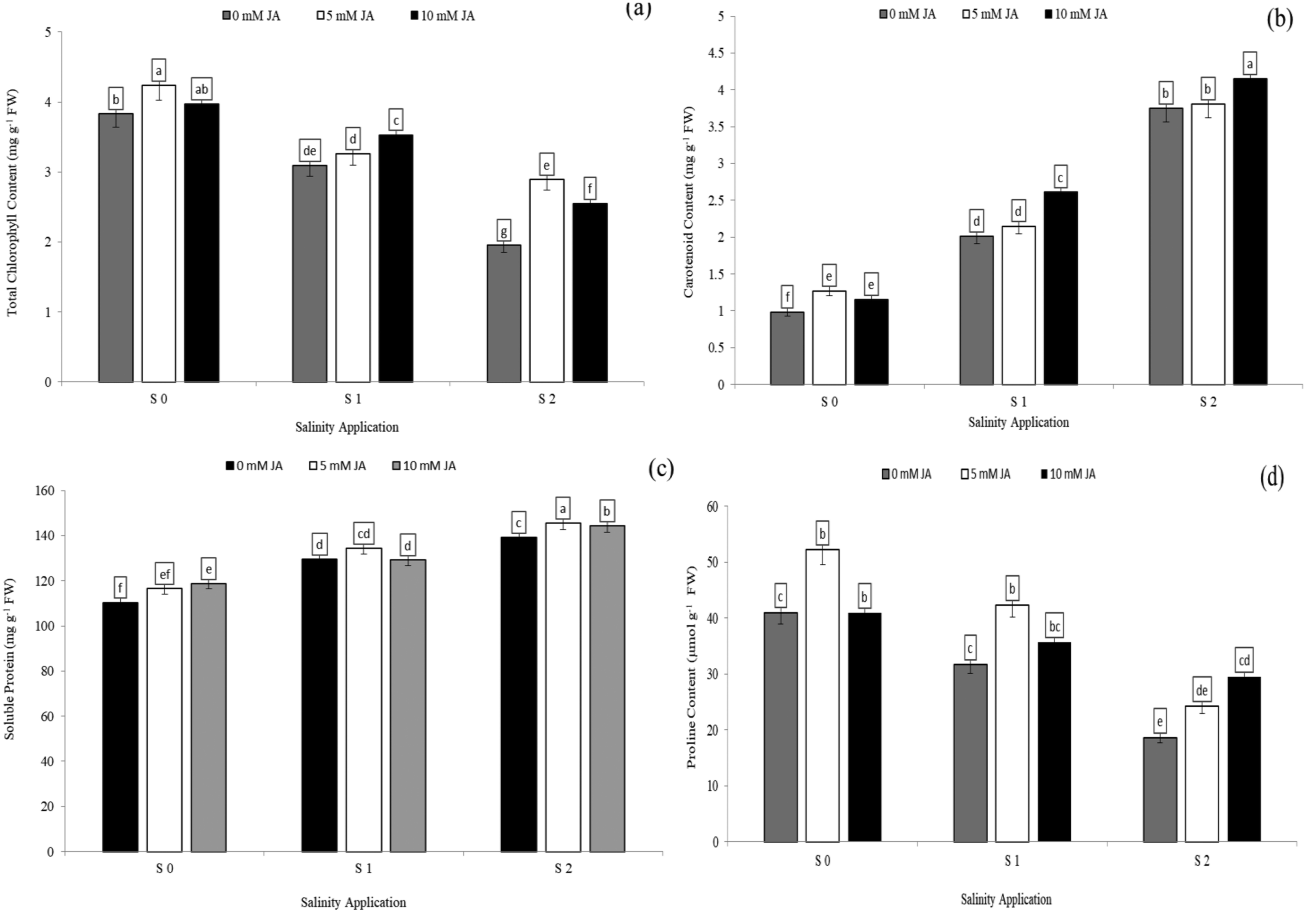
The interaction between salinity and jasmonic acid on total chlorophyll content, carotenoid content, soluble protein content and proline content. Total chlorophyll content (A), carotenoid content (B), soluble protein (C) and proline content (D) of forage sorghum (*Sorghum bicolor* (L.) Moench) variety Abu Sabeein as effluence by the interaction between different salinity levels S0 = 0, S1 = 2 and S2 = 4 g NaCl kg^−1^ dry soil and different jasmonic acid levels. Data were averaged over two growing seasons. The values of each trait labeled by different letters indicate significant differences separated by the LSD test (*P* < 0.05).

Soluble protein and proline content were significantly decreased with increased salinity rates. In the interaction between salinity and jasmonic acid, at the control of jasmonic acid (0 mM JA), the high salinity rate of S2 reduced the proline content by 54.6% (Fig. 4C), in comparison to control of salinity (S0) at the control of jasmonic acid (0 mM JA). Moreover, at the same rate of jasmonic acid (0 mM JA), the high salinity rate of S2 increased soluble protein content by 26.2% (Fig. 4D). Jasmonic acid improved the soluble protein, proline content, chlorophyll content and carotenoid content. According to the results of the interaction between salinity and jasmonic acid, at high saline rate of S2, 5 mM JA level increased the chlorophyll content and soluble protein content by 48.2% (Fig. 4A) and 4.5% (Fig. 4C) respectively, in comparison with control of salinity. At the same salinity rate, the level of 10 mM JA were increased the carotenoid content by 10. 7% (Fig. 4B), and proline content by 58.5% (Fig. 4D) as compared with the 10 mM JA at the same salinity rate.

### Growth parameters and photosynthetic pigments as affected by the combination of jasmonic acid and humic acid

The growth parameters (plant height and relative growth rate) and photosynthetic pigments (chlorophyll content and carotenoid) were affected by the interaction between jasmonic acid and humic acid (Table 3). According to the results of the interaction between jasmonic acid and humic acid, the treatment with the high rate of humic acid of HA2 and with 5 mM JA achieved the highest plant height (263.6 cm) and relative growth rate (5.3). However, the treatment of HA1 + 5 mM JA were recorded the highest chlorophyll content (3.53 mg g^−1^ FW) and carotenoid content (1.77 mg g^−1^ FW) (Table 5).

**Table 5:** Interaction between humic acid and jasmonic acid on soluble protein content and most antioxidants enzyme activities traits. The average of two season of plant height, relative growth rate, chlorophyll content and carotenoid content of forage sorghum (*Sorghum bicolor* (L.) Moench) variety Abu Sabeein as effluence by the interaction between different jasmonic acid levels (0, 5 and 10 mM JA as a foliar application) with different humic acid rates (0 (HA0), 3 (HA1), and 6 g HA kg^−1^ dry soil (HA2)).

Humic acid rates	Jasmonic acid levels	Plant height (cm)	Relative growth rate	Chlorophyll content (mg g^-1^ FW)	Carotenoid content (mg g^−1^ FW)
HA 0	0 mM	193.39 ± 18.92e	2.79 ± 0.43ed	2.33 ± 0.43e	0.96 ± 0.14ef
	5 mM	226.93 ± 20.47d	4.54 ± 0.78b	2.77 ± 0.56d	1.14 ± 24cd
	10 mM	178.90 ± 27.46f	3.91 ± 0.54c	2.80 ± 0.38cd	1.13 ± 0.16cd
HA1	0 mM	231.38 ± 29.16d	2.63 ± 0.41d	2.81 ± 0.74cd	0.81 ± 0.14f
	5 mM	227.99 ± 32.64d	3.79 ± 0.98c	3.53 ± 0.23a	1.77 ± 1.25a
	10 mM	192.41 ± 22.67e	2.86 ± 0.21d	3.03 ± 0.64bc	1.23 ± 0.24c
HA2	0 mM	254.30 ± 27.99b	3.94 ± 0.85c	2.74 ± 0.59d	1.00 ± 0.18de
	5 mM	263.56 ± 22.05a	5.28 ± 0.65a	2.99 ± 0.42bc	1.51 ± 0.33b
	10 mM	240.37 ±27.68c	4.99 ± 0.65ab	3.21 ± 0.59b	1.20 ± 0.20c

**Note:**

All analyzed data are expressed as mean ± SD values of three biological replicates per treatment. Within the same parameter, means followed by different letters are statistically different at the 0.05 probability level. Means separated by the LSD test.

### Antioxidants enzyme activities as affected by the interaction between salinity and humic acid

**Figure 5:**
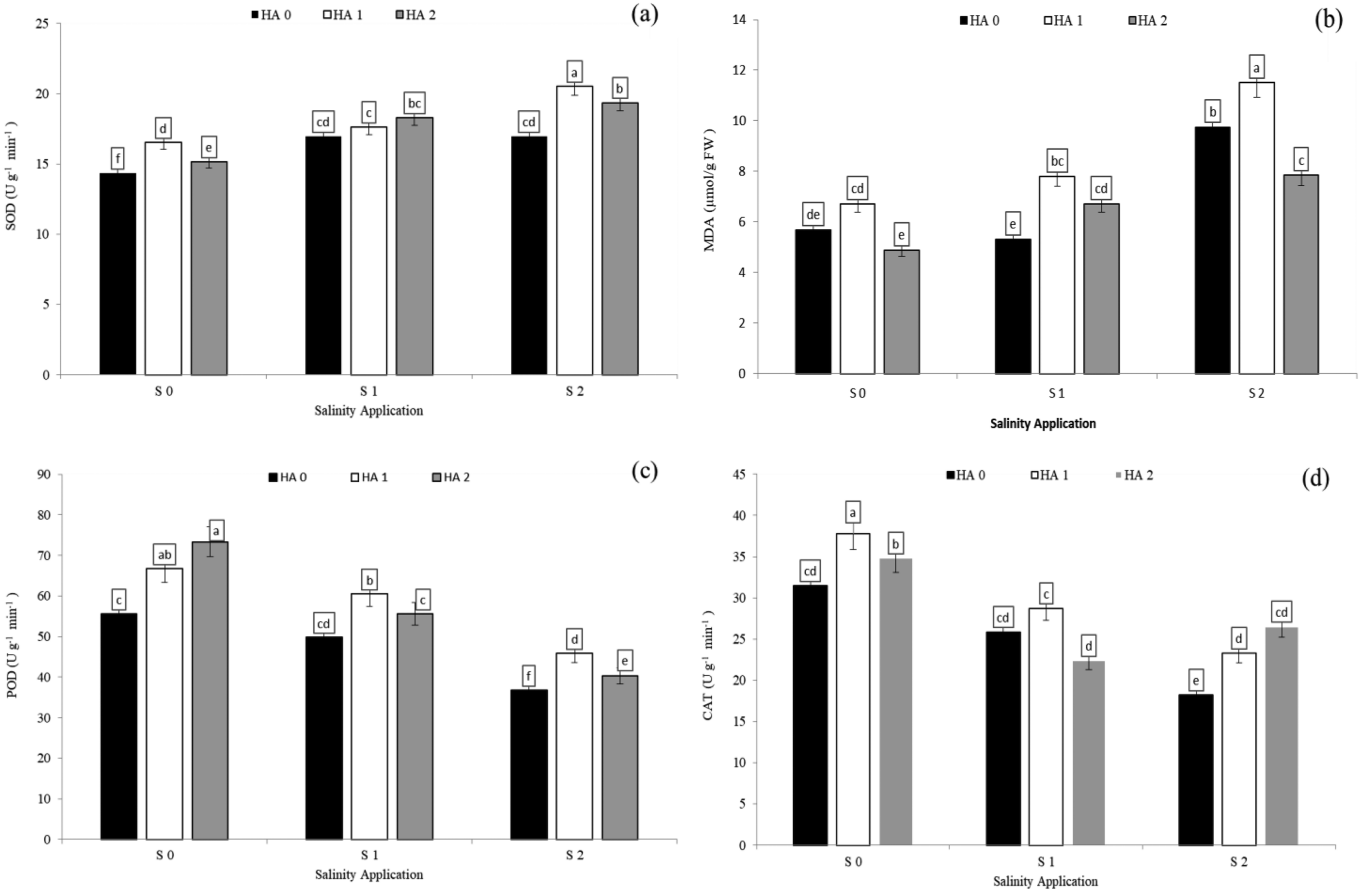
The interaction between salinity and jasmonic acid on SOD, MDA, POD and CAT activities. Superoxide dismutase (SOD) (A), malondialdehyde (MDA) (B), peroxidase (POD) (C) and catalase (CAT) (D) activities of forage sorghum (*Sorghum bicolor* (L.) Moench) variety Abu Sabeein as effluence by the interaction between different salinity levels (S0 = 0, S1 = 2 and S2 = 4 g NaCl kg^−1^ dry soil) and different humic acid rates (HA0 = 0, HA1 = 3 and HA2 = 6 g HA kg^−1^ dry soil). Data were averaged over two growing seasons. The values of each trait labeled by different letters indicate significant differences separated by the LSD test (*P* < 0.05).

Salinity stress significantly reduced antioxidant enzyme activities except SOD activity and MDA content. However, increased salinity rate significantly increased SOD activity and MDA content. In the interaction between salinity and humic acid, at the control of humic acid (HA0), high salinity rate of S2 decreased POD activity by 33.8% (Fig. 5A), CAT by 40.1% (Fig. 5D), and APX by 36.4% (Table 4) over the control (S0) at HA0. Moreover, at the same rate of humic acid (HA0), as compared with S0, S2 increased SOD activity from 14.3 to 17.0 U g^−1^ min^−1^ (Fig. 5A) and MDA content from 5.7 to 9.8 µmol/g FW (Fig. 5B). The activities of antioxidant enzymes were enhanced and increased by humic acid application. At S2 salinity rate, the medium rate of humic acid of HA1 increased SOD activity by 20.9% (Fig. 5A), MDA content by 18.0% (Fig. 5B), POD activity by 24.6% (Fig. 5C), and APX activity by 21.7 (Table 4), in comparison to control of humic acid (HA0). At the same salinity level of S2, the HA2 treatment increased the CAT activity by 45.5% (Fig. 5D) over the control of humic acid at the same salinity.

### Antioxidants enzyme activities as affected by interaction between salinity and jasmonic acid

**Figure 6:**
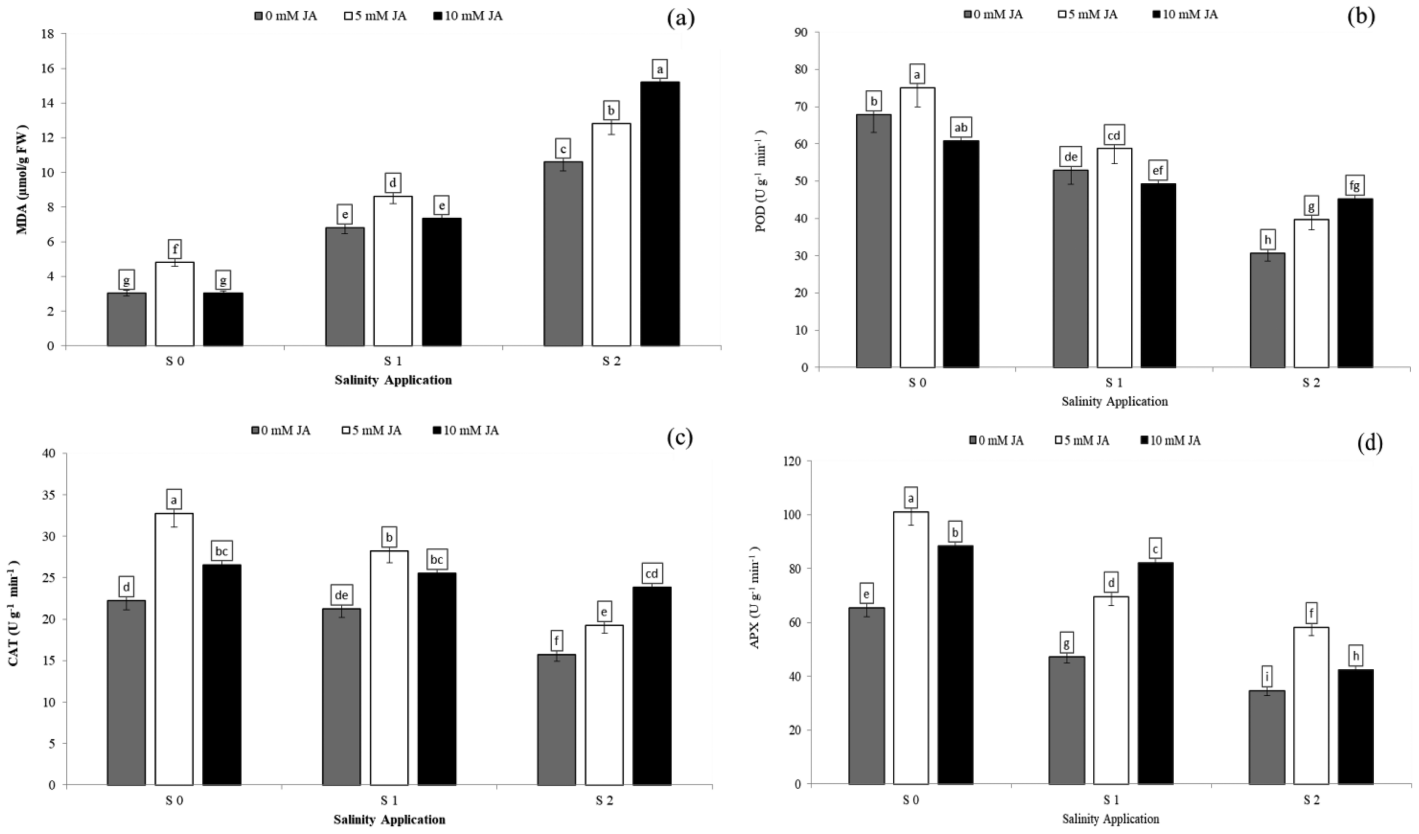
The interaction between salinity and jasmonic acid on MDA, POD, CAT and APX activities. Malondialdehyde (MDA) (A), peroxidase (POD) (B), catalase (CAT) (C) and ascorbate peroxidase (D) (APX) activities of forage sorghum (*Sorghum bicolor* (L.) Moench) variety Abu Sabeein as effluence by the interaction between different salinity levels S0 = 0, S1 = 2 and S2 = 4 g NaCl kg^−1^ dry soil and different jasmonic acid levels. Data were averaged over two growing seasons. The values of each trait labeled by different letters indicate significant differences separated by the LSD test (*P* < 0.05).

Regarding the combination between salinity and jasmonic acid, both jasmonic acid levels improved the antioxidants enzyme activities. At the S2 salinity rate, 5 and 10 mM jasmonic acid increased MDA content by 20.7% and 43.2% respectively (Fig. 6A), POD activity by 29.7% and 47.5% respectively (Fig. 6B), CAT activity by 22.6% and 51.8% respectively (Fig. 6C), and APX activity by 67.8% and 22.6% respectively (Fig. 6D) in comparison with control of jasmonic acid (0 mM JA) at high salinity rate. At the S1 salinity level, 5 mM JA were more effective on the antioxidants enzyme activities except for APX activity.As compared with 0 mM JA at the S1 salinity level, 5 mM JA increased MDA content from 6.8 to 8.6 µmol/g FW (Fig. 6A), POD activity from 52.9 to 58.8 U g^−1^ min^−1^ (Fig. 6B), and CAT activity from 21.2 to 28.2 U g^−1^ min^−1^ (Fig. 6C), while 10 mM JA increased the APX activity from 6.8 to 8.6 U g^−1^ min^−1^ (Fig. 6D).

### Soluble protein and antioxidants enzyme activities as affected by the combination of jasmonic acid and humic acid

Soluble protein and antioxidants enzyme activities such as SOD, POD and CAT activities were significantly affected by the combination between jasmonic acid and humic acid (Table 3). In the interaction between jasmonic acid and humic acid, sorghum plants treated with HA1 + 5 mM JA achieved the highest soluble protein content (147.4 mg g^−1^ FW) and CAT activity (40.6 U g^−1^ min^−1^). However, sorghum plants treated with HA2 + 10 mM JA recorded the highest SOD activity (25. 7 U g^−1^ min^−1^) and POD activity by 66.7 U g^−1^ min^−1^. On the other hand, the treatment of JA0 + HA0 recorded the lowest content of soluble protein and the lowest activities of most antioxidants enzymes (Table 6).

**Table 6:** The interaction between different jasmonic acid levels (0, 5 and 10 mM JA as a foliar application) with different humic acid rates (0 (HA0), 3 (HA1), and 6 g HA kg^−1^ dry soil (HA2)). The average of two season of soluble protein and most antioxidants enzyme activities of forage sorghum (*Sorghum bicolor* (L.) Moench) variety Abu Sabeein as effluence by the interaction between different jasmonic acid levels (0, 5 and 10 mM JA as a foliar application) with different humic acid rates (0 (HA0), 3 (HA1), and 6 g HA kg^−1^ dry soil (HA2)).

Humic acid rates	Jasmonic acid levels	Soluble protein (mg g^−1^ FW)	SOD (U g^−1^ min^−1^)	POD (U g^−1^ min^−1^)	CAT (U g^−1^ min^−1^)
HA 0	0 mM	103.0 ± 07.61g	13.52 ± 0.97g	34.93 ± 08.44f	19.45 ± 1.32g
	5 mM	110.9 ± 04.22f	16.52 ± 1.74f	43.43 ± 10.50e	26.38 ± 1.91de
	10 mM	118.9 ± 05.87e	17.38 ± 2.37ef	58.70 ± 21.19c	21.27 ± 3.53fg
HA1	0 mM	125.8 ± 06.26cd	18.58 ± 2.47de	50.55 ± 09.90cd	23.94 ± 3.90ef
	5 mM	147.4 ± 14.88a	20.59 ± 2.32c	62.78 ± 20.85ab	40.57 ± 10.79a
	10 mM	139.1 ± 22.39b	19.13 ± 2.55 d	50.95 ± 13.77cd	32.78 ± 5.92c
HA2	0 mM	127.6 ± 11.04cd	19.65 ± 3.40c	62.78 ± 11.66b	27.77 ± 3.30d
	5 mM	132.0 ± 07.75c	23.15 ± 2.49b	65.53 ± 21.49ab	35.57 ± 7.29bc
	10 mM	123.5 ± 07.26de	25.65 ± 3.40a	66.68 ± 23.92a	36.90 ± 7.50b

**Note:**

All analyzed data are expressed as mean ± SD values of three biological replicates per treatment. Within the same parameter, means followed by different letters are statistically differentat the 0.05 probability level. Means separated by the LSD test.

## Discussion

When plants are grown under saline stress conditions and during the elongation process of new plant cells, the excess of salts modifies the cell wall’s metabolic activities, causing the deposition of various materials that limit cell wall elasticity. Cell walls become rigid, and consequently, the turgor pressure efficiency in cell enlargement decreased (Ali et al., 2004). The management of saline soils is considered a major challenge due to its unfavorable physical and chemical properties and low soil microbial activity, which lead to a reduction in soil quality and crop productivity (Ali et al., 2021, 2022).

### Growth parameters

In the current study, NaCl salinity stress significantly inhibited plant growth traits of forage sorghum, including plant height, leaf area, and relative growth rate. The inhibition in the growth parameters may be due to osmotic impacts of salt stress or increments in growth retardants, water imbalance (Taha et al., 2020), ions toxicity, decreased nutrient absorption, reduced internode elongation and length (Ali et al., 2022), and formation of new apical tissues (Ibrahim et al., 2016b). The contrary results were reported by Qados (2011) noted that the plant height significantly was increased at the low salinity levels. However, similar findings were reported by Taffouo et al. (2010) on cowpea (*Vigna unguiculata* L.), Ali et al. (2021) on sorghum seedling, and Taha et al. (2021b) on wheat plant.

In this study, leaf area was decreased with increasing salinity stress. The decrease in leaf area under salinity stress has been attributed to suppressed cell division, the shrinkage of the cell contents leading to reduced development and differentiation of tissues, unbalanced nutrition, and disturbed avoidance mechanism (Katerji et al., 2003). In this regard, Taha et al. (2020) reported that under saline conditions all growth characteristics including leaf area of soybean plants were significantly lower than those of plants grown in normal soils. The decrease in leaf area has primarily been a result of the inhibition of owing to Na accumulation, cell growth and division (Al Ashkar et al., 2020). Our findings agreed with those of Omer & Abdalla (2017) and Badawy et al. (2021), who reported a negative correlation between salinity and leaf area.

Relative growth rate depends on canopy photosynthesis per area of land. In the present study, the relative growth rate of salt-stressed plants in the high salinity treatment was lower than that of the other salinity treatments. Decreases in plant growth under salinity stress might be endorsed to the reduction in water absorption due to reduced osmotic pressure in the soil solution (Taha et al., 2021a). Our results disagreed with those of Rasmuson & Anderson (2002) who mentioned that the salinity stress increased the relative growth rate. These results confirm by Seleiman et al. (2020) reported that relative growth rate was markedly decreased under high NaCl salinity stress.

Plant hormones play essential roles in stress responses and adaptation. It is clearly defined that jasmonic acid (JA) increased in response to salinity (Verma, Ravindran & Kumar, 2016). In this study, growth parameters were increased with the application of jasmonic acid (JA) and humic acid (HA) under both saline and non-saline conditions. This can be due to the mitigation of adverse effects of salinity stress on plant height, leaf area and relative growth rate when JA and HA were applied (Ali et al., 2020). This results agreed with Alavi-Samani, Kachouei & Pirbalouti (2015) who reported that JA application recorded the highest plant height and leaf area at the high salinity stress.

Organic fertilizer application such as vermicompost and humic acid were optimal for improving soil properties because of the increased organic matter, nutrient contents, the vital role of soil enzyme action, improved soil aeration, and enhanced microbial action in soil, thus improving soil physical and chemical properties (Ding et al., 2021). Humic acid (HA) can improve plant growth by increasing cell membrane permeability, which can promote water absorption and nutrients uptake (Ali et al., 2020). In this study, the results revealed that, under different salinity levels, soil amendment with humic acid improved growth traits such as plant height, leaf area, and relative growth rate under salinity stress conditions. The increase in the plant height in the HA amended treatments was most probably due to the root zone improved by humic acid (Ding et al., 2021). Similar impacts were shown by Heidari & Minaei (2014), Ghorbani et al. (2010), and Haghighi, Saki-Nejad & Lack (2011), who reported that HA application had remarkable effects on vegetative growth and increased plant height, relative growth rate, leaf area and photosynthetic activity.

### Total chlorophyll content and carotenoid content

The chlorophyll content is widely used as an index to indicate the abiotic tolerance level in plants. Protection of chloroplast and photosynthetic machinery is the first target of defense under stressful conditions (Anjum et al., 2011). In this study, total chlorophyll content decreased with increasing salinity levels. The negative impact of high NaCl salt stress on chlorophyll content might be caused by inhibited nutrient uptake (Seleiman et al., 2020), inhibited biosynthesis of chlorophyll, and increased chlorophyll-degrading enzyme chlorophyllase (Taha et al., 2021a). Similar results were showed by Latif & Mohamed (2016) in common bean and Akladious & Mohamed (2018) in the pepper plants. The reduction in chlorophyll content under salt stress may be due to the reduction in carbon use efficiency and uptake of minerals such as Mg and Fe (Latif & Mohamed, 2016).

In the present study, jasmonic acid application increased the total chlorophyll content and carotenoid content under saline conditions, which is one of the factors contributing to higher photosynthetic of forage sorghum plants under salinity conditions. Our results were similar to those of Sofy et al. (2020), who mentioned that the application of JA improved the accumulation of photosynthetic pigments under abiotic stress conditions. This may be due to the protective role of JA, which can enhance photosynthesis and the absorption of important minerals under abiotic stress. These results suggested that exogenous JA treatment could alleviate salinity stress, allowing plants to increase their tolerance to unfavorable conditions.

The stimulation in photosynthetic pigments caused by humic acid caused may be due to the decrease of pH value and increase in the activity of soil organisms which release more nutrients from the soil such as Fe (Latif & Mohamed, 2016). In the present study, under salinity conditions, application of humic acid improved the total chlorophyll content and carotenoid content. These results have been confirmed by Akladious & Mohamed (2018) and Kaya et al. (2018). It has also been confirmed that HA application can enhance photosynthesis activity like chlorophyll and increase tolerance in stress conditions by increasing the enzyme rubisco (Latif & Mohamed, 2016).

### Soluble protein and proline content

Improving soluble protein and proline content are an important mechanism that plants use to alleviate the harmful effect of salinity stress (Hatami et al., 2018). In the present investigation, soluble protein was increased at the high salinity level and subsequently decreased at the control of salinity level. This result were contradicted with the results of Ibrahim et al. (2018) and Dustgeer et al. (2021) who noted that under salinity stress, the soluble protein was significantly reduced. The reduction in soluble protein under salinity may be due to decreased potassium content, reduction in sodium content, protease enzyme activity, and hydrolysis of the rubisco enzyme (Hatami et al., 2018). In the present study, proline content was decreased by increasing salinity levels. This result agreed with the results of Zrig et al. (2015) and Nimir et al. (2015), but disagreed with the findings of Qiu et al. (2014) who reported that salinity stress significantly increased the proline content. The varied changes in the downregulation and upregulation of the contents of soluble protein and proline in different studies are caused by many reasons, including different crops studied, genetic variance in the same crops, and the levels of salt applied.

In the study, humic acid improved the contents of soluble protein and proline of forage sorghum. Our results were dissimilar with Farahat, Mazhar & Mahgoub, (2012) who reported that humic acid significantly decreased proline content. However, our results are same as Kumari et al. (2006) who reported that humic acid improved the proline content. The increase in proline content might be due to a rapid accumulation of a specific protein set. In the present investigation, HA improved the protein content. Similar findings were reported by Fernández et al. (2013) and Hatami et al. (2018).

Jasmonic acid can protect the plant from toxicity ions in the different stages by managing the antioxidant machinery and synthesis of proteins (Sirhindi et al., 2015). In the study, jasmonic acid increased the contents of soluble protein and proline of forage sorghum. An increased in proline contents in comparison with the control by jasmonic acid was also reported by Ali et al. (2020). However, different results were noted by Farhangi & Ghassemi (2018) which reported that jasmonic acid reduced proline content under salt stress in the wheat.

### Superoxide dismutase and malondialdehyde contents

Superoxide dismutase (SOD) is one of the enzymes responsible for eliminating O_2_^−^ and is considered an essential antioxidant in cells. In our study, high salinity stress increased SOD activity and MDA content. The increased in SOD activity coincided with an increasing in the activities of Mn-SOD and Fe-SOD (Anjum et al., 2011). These results were contrary to Sekmen et al. (2012) who reported that the SOD activity was decreased under salinity stress in *Gypsophila oblanceolate* plant. Similar results were noticed by Ali et al. (2020), and Nimir et al. (2015) who suggested that under soil saline conditions, MDA content was substantially increased by increasing soil salinity.

Our study showed that significant increases in SOD activity and MDA content were observed in forage sorghum plants treated with JA. The increase in SOD activity agreed with the results of Anjum et al. (2011), Noriega et al. (2012) and Ali et al. (2019) who reported that exogenous JA application significantly improved SOD activity and MDA content under salinity stress. However, the increase in MDA content under salinity stress by jasmonic acid differed from the findings of Qiu et al. (2014).

In our results, the application of HA under saline condition increased SOD activity and MDA content. Different result was showed by Kaya et al. (2018) who reported that HA reduced the activity of SOD in the maize plant under NaCl salinity stress. Similar results were reported by Kesba & El Beltagi (2012) who found that HA increased the antioxidant enzyme activity, including SOD activity in response to salinity stress. Similar results were reported by Gautam & Singh (2011) who found that MDA content under NaCl-stressed plants was increased significantly by applying humic acid. Ali et al. (2019) noted that humic acid and jasmonic acid application increased antioxidant enzyme activities including CAT, POD and SOD in sorghum seedling.

### Catalase and peroxidase activity

Enhancement of the activities of antioxidative enzymes in plants under saline conditions could improve the protecting mechanism to decrease adverse impacts by salt stress. In this study, soil saline stress caused reductions in CAT and POD activities. Reduced CAT activity under salinity stress might have promoted H_2_O_2_ accumulation, which could result in a Haber–Weiss reaction from hydroxyl radicals (Ali et al., 2021). The decrease in POD and CAT activities in our study confirmed with the results of Qiu et al. (2014) and Sekmen et al. (2012). However, a disconfirmed result has been shown by Ibrahim et al. (2018) and Ibrahim et al. (2016a) on wheat plants treated with soil salinity, reporting that POD and CAT activities increased under NaCl stress.

In this study, foliar application of jasmonic acid improved CAT and POD activities in the leaves of forage sorghum plants under soil saline conditions. Our result was similar to the findings of Anjum et al. (2011), who suggested that the wheat and soybean plants exposed to salinity and treatment with JA application significantly increased the antioxidant enzyme activities including CAT and POD and played an essential role in antioxidant defense required for salt tolerance. In addition, Farhangi & Ghassemi (2018) noted that the treatment of soybean plants under salt stress by salicylic acid and jasmonic acid promoted the antioxidant enzyme activities. In this study, the application of HA improved plant defense systems such as POD and CAT in the forage sorghum plants, and the both rates increased POD and CAT activities. Many studies have also noted that the POD and CAT activities increased by application of HA, and it efficient in improving tolerance of salt in cucumber (Karakurt et al., 2015), date palm (*Phoenix dactylifera* L.), and hot pepper (Aminifard et al., 2012). However, our result differed from the findings of Kaya et al. (2018) who found that the humic acid application decreased CAT and POD activities under salt stress. Ali et al. (2019) reported that CAT and SOD activities increased after application of jasmonic acid and humic acid under salt stress.

### Ascorbate peroxidase activity

Under salt stress, the APX plays an essential role in protecting plants by reducing H_2_O_2_ (Anjum et al., 2011). Our study showed that increasing salinity level reduced APX activity. Similar result was reported by Qiu et al. (2014), Ali et al. (2021) and Sekmen et al. (2012). However, different results were noted by Ozdener & Kutbay (2008) and Farhangi & Ghassemi (2018) who reported that salinity stress reduced APX activity. In the aforementioned results, the application of jasmonic acid and humic acid enhanced the antioxidant enzyme activities including APX activity under salinity condition. Increased APX activity by HA application has been reported in maize plants exposed to salinity (Kaya et al., 2018). Kesba & El Beltagi (2012) also reported that HA improved APX activity response to salinity stress. Our results showed that exogenous JA significantly increased APX activity. Ali et al. (2019) reported that APX and SOD activities were increased by the application of jasmonic acid and humic acid under salt stress. Our work was in line with the findings of Kaya & Doganlar (2016), who suggested that exogenous JA increased APX activity in tobacco (*Nicotiana tabacum* L.) plants under salt stress. Also, Piotrowska et al. (2009) report that APX activity we increased in the plants treated with JA exposed to Pb stress. Moreover, exogenous JA induced the synthesis of antioxidant metabolites that provided additional resistance to neutralize the toxic effects of ROS generated by salt stress (Qiu et al., 2014).

## Conclusions

Our study examined the effects of different rates of humic acid as soil amendment and jasmonic acid as foliar spray on growth parameters, total chlorophyll content, carotenoid content and antioxidant enzyme activities of forage sorghum exposed to salinity. High soil salinity rate of 4 g NaCl kg^−1^ soil decreased all the parameters tested, except for protein content, SOD activity, and MDA content. Jasmonic acid significantly improved salt stress tolerance in forage sorghum plants; and the plants sprayed with 10 mM JA level had higher POD and SOD activities. Among different humic acid rates, 3 g HA kg^−1^ dry soil successfully increased all the parameters tested. Generally, 10 mM JA and 3 g HA kg^−1^ dry soil produced the best positive effects on forage sorghum plants physiological responses. Our study suggested that proper management of humic acid as soil amendment and jasmonic acid as foliar spray could be conducted in salt-affected soils to sustain forage growth and increase crop yield and productivity of forage sorghum.

## Supplemental Information

10.7717/peerj.13793/supp-1 Supplemental Information 1 All the data of the parameters.

## Additional Information and Declarations

### Data Availability

The following information was supplied regarding data availability: The raw data are available in the Supplemental File.
